# Novel *POLG* variants associated with late-onset de novo status epilepticus and progressive ataxia

**DOI:** 10.1212/NXG.0000000000000181

**Published:** 2017-08-09

**Authors:** Yi Shiau Ng, Helen Powell, Nigel Hoggard, Doug M. Turnbull, Robert W. Taylor, Marios Hadjivassiliou

**Affiliations:** From the Wellcome Centre for Mitochondrial Research (Y.S.N., H.P., D.M.T., R.W.T.), Institute of Neuroscience, Newcastle University, Newcastle upon Tyne; and Sheffield Teaching Hospitals NHS Trust and University of Sheffield (N.H., M.H.), Royal Hallamshire Hospital, Sheffield, United Kingdom.

Mitochondrial disease is phenotypically and genetically heterogeneous with an estimated prevalence of 1 in 4,300.^1^ Mutations in the *POLG* gene, encoding the catalytic subunit of DNA polymerase gamma, are an important cause of mitochondrial disease. The spectrum of clinical manifestations in *POLG*-related mitochondrial disease is variable,^2^ with disease onset ranging from adulthood-onset dominant or recessive progressive external ophthalmoplegia (chronic progressive external ophthalmoplegia), ataxia-neuropathy spectrum, myoclonic epilepsy, myopathy, and sensory ataxia to childhood-onset Alpers syndrome, which is characterized by intractable seizures, psychomotor regression, and hepatic impairment. Epilepsy is a poor prognostic factor in *POLG* mutations,^3^ and the onset of epilepsy often clusters in childhood (<5 years) and teenage.^4^ However, late-onset epileptic encephalopathy is uncommon.^4,5^ Herein, we describe a patient who died of de novo, late-onset refractory status epilepticus with the identification of 2 novel variants in the *POLG* gene.

## Case report.

A 69-year-old woman presented with an 8-year history of slowly progressive gait ataxia associated with dysarthria to the regional ataxia center. She also noted to have generalized myoclonic jerks for 9 months. There was no other medical history or relevant family history of any neurologic disorder. On examination, she had evidence of ophthalmoplegia in all directions of gaze. She was found to have prominent gait and lower limb ataxia. Myoclonus was demonstrable with outstretched arms. Reflexes were present and symmetrical. She was just able to walk with a stick and required 1 person's assistance. Mitochondrial disease was suspected, and she underwent a muscle biopsy.

She was admitted acutely to the hospital following 2 episodes of generalized tonic-clonic seizures at age 71. She was treated with IV phenytoin and levetiracetam. Her management was rapidly escalated to the administration of general anesthesia due to convulsive status epilepticus. Laboratory investigations including routine biochemistry, autoantibodies, septic screens, and CSF analysis were unremarkable, except a slightly raised serum lactate level at 3.3 mol/L (normal: <2.2 mmol/L). EEG showed encephalopathic changes. MRI head T2 and fluid attenuation and inversion recovery sequences revealed stroke-like lesions (figure, A), in addition to the previously documented changes in the clinic (figure, B). Epilepsia partialis continua, affecting the left face, arm, and leg, emerged on day 12 of admission. Her seizures were suprarefractory to treatment, despite receiving a combination of phenytoin, levetiracetam, clonazepam, propofol, midazolam, and pulse methylprednisolone. She died of worsening epileptic encephalopathy and multiorgan failure after 2 weeks of hospitalization.

**Figure:**
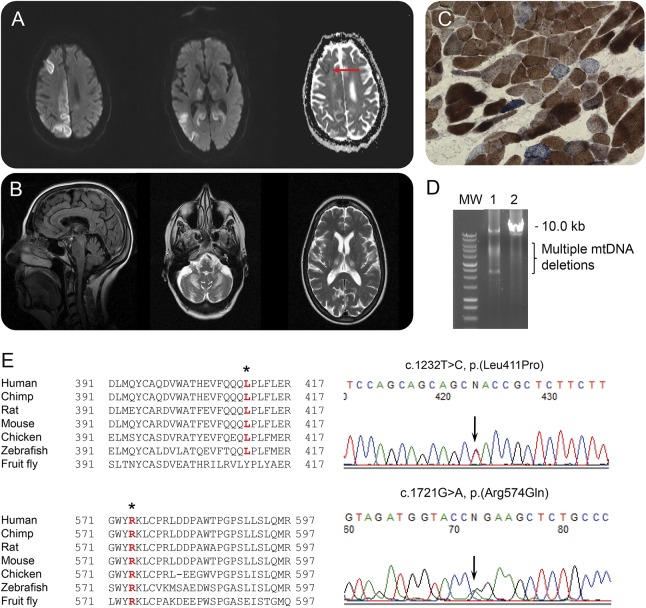
Neuroimaging, muscle biopsy, and molecular genetic findings (A) Head MRI performed at age 71 following admission in status epilepticus. Diffusion-weighted imaging sequence showed restricted diffusion in occipital, parietal, and frontal lobes, thalami, and with low ADC map in the right frontal lobe (red arrow). (B) Head MRI performed at onset of ataxia aged 69. Sagittal T1 view showed cerebellar atrophy and axial T2 view showed symmetrical hyperintensities in the cerebellar dentate nuclei and thalami. (C) Sequential cytochrome *c* oxidase (COX)–succinate dehydrogenase histochemistry demonstrates a mosaic distribution of COX-deficient muscle fibers (blue) among fibers exhibiting normal COX activity (brown). (D) Long range PCR amplification of muscle DNA across the major arc confirms multiple mitochondrial DNA (mtDNA) deletions in patient muscle (lane 1) compared with age-matched control muscle (lane 2); MW = molecular weight marker. (E) Alignments of mutation-containing *POLG* regions across multiple species show the evolutionary conservation of the heterozygous c.1232T>C, p.(Leu411Pro) and c.1721G>A, p.(Arg574Gln) missense *POLG* variants. The c.1232T>C, p.(Leu411Pro) variant is absent from both the ExAC browser (exac.broadinstitute.org)and the NHLBI ESP (evs.gs.washington.edu/EVS/) database (both accessed on October 6, 2016), thus representing a novel missense change, while the c.1721 G>A, p.(Arg574Gln) variant has only identified in 3/120480 alleles on the ExAc browser. A different *POLG* variant affecting the same amino acid c.1720C>T, p.(Arg574Trp) has been previously reported in trans with other known pathogenic variants in 4 unrelated patients, according to the Human DNA Polymerase Gamma Mutation Database (tools.niehs.nih.gov/polg/index.cfm/main/search) (accessed on May 17, 2017). Affected amino acids are highlighted by an asterisk; sequence identity is shown by bold, red typeface.

This patient was tested negative for common mitochondrial DNA (mtDNA) point mutations, including m.3243A>G, m.8344A>G, and m.8993T>C/G. She was also tested negative for 3 common *POLG* mutations (p.Ala467Thr, p.Trp748Ser, and p.Gly848Ser). Her muscle biopsy revealed histochemical and molecular genetic evidence of mitochondrial dysfunction, including cytochrome *c* oxidase–deficient fibers (figure, C) and variable mtDNA deletions (figure, D). No pathogenic variant was identified in *TWNK* and *RRM2B*. Direct sequencing of the *POLG* gene (GenBank accession number NM_002693.2) identified 2 rare variants, c.1232T>C, p.(Leu411Pro) and c.1721G>A, p.(Arg574Gln), both affecting conserved amino acids and predicted to be damaging (figure, E). Familial segregation studies were not feasible, as she was the only child and both her parents were deceased.

## Discussion.

Our patient's initial presentation of a progressive cerebellar ataxia plus other neurologic features including external ophthalmoplegia and myoclonus is highly suggestive of a mitochondrial etiology. Moreover, her neuroimaging findings of bilateral signal abnormalities in thalami, cerebellar dentate nuclei, and cerebellar atrophy have previously been reported in *POLG*-related mitochondrial disease.^6^ However, the development of fatal epileptic encephalopathy is rather surprising, given the insidious onset of her illness. Our case highlights the progressive nature of *POLG*-related mitochondrial disease, the overlap of clinical syndromes and difficulty of predicting the trajectory of disease progression, and the management challenge of refractory mitochondrial epilepsy.^4^ The presence of focal onset motor status, together with the acute stroke-like lesions, is likely related to the neuronal energy failure^6^ of which inhibitory interneurons have been shown to be particularly vulnerable to mitochondrial dysfunction.^7^

We were unable to unequivocally conclude whether these 2 variants were in cis or in trans. We speculate that our patient had a late-onset recessive *POLG* disease, given that recessive *POLG* disease is more common than dominant presentations according to our experience and reported cases in the literature. Both variants are located in the linker domain of POLG, and we have recently showed that mutations (homozygous or compound heterozygous) in this region are associated with later disease presentation and longer survival compared with other domains within the POLG protein.^4^

We propose that *POLG*-related mitochondrial disease should be a differential diagnosis in cases of de novo status epilepticus, particularly with other clinical features such as ataxia and external ophthalmoplegia, irrespective of age. Full sequencing of *POLG* should be performed because more than 20% of patients do not carry 1 of the 3 common mutations,^4^ as exemplified by this case.
